# Multidrug-resistant organism-peritoneal dialysis associated peritonitis: clinical and microbiological features and risk factors of treatment failure

**DOI:** 10.3389/fmed.2023.1132695

**Published:** 2023-05-10

**Authors:** Shizheng Guo, Liming Yang, Xueyan Zhu, Xiaoxuan Zhang, Lingfei Meng, Xinyang Li, Siyu Cheng, Xiaohua Zhuang, Shengmao Liu, Wenpeng Cui

**Affiliations:** ^1^Division of Nephrology, The Second Hospital of Jilin University, Changchun, China; ^2^Division of Nephrology, First Hospital of Jilin University – The Eastern Division, Changchun, China; ^3^Division of Nephrology, Jilin Central Hospital, Jilin, China; ^4^Division of Nephrology, Jilin FAW General Hospital, Changchun, China

**Keywords:** peritoneal dialysis, peritonitis, multi-drug resistant, antimicrobial sensitivity pattern, risk factors

## Abstract

**Background:**

Multidrug-resistant (MDR) bacterial infection causes difficulty in the therapy of peritoneal dialysis-associated peritonitis (PDAP); however, there are few studies on multidrug-resistant organism (MDRO)-PDAP. In view of growing concerns about MDRO-PDAP, the aim of this study was to investigate the clinical features, risk factors of treatment failure, and causative pathogens of MDRO-PDAP.

**Methods:**

In total, 318 patients who underwent PD between 2013 and 2019 were included in this multicenter retrospective study. Clinical features, patient outcomes, factors related to treatment failure, and microbiological profiles associated with MDRO-PDAP were analyzed and risk factors for treatment failure associated with MDR-*Escherichia coli (E. coli)* were further discussed.

**Results:**

Of 1,155 peritonitis episodes, 146 eligible episodes of MDRO-PDAP, which occurred in 87 patients, were screened. There was no significant difference in the composition ratio of MDRO-PDAP between 2013–2016 and 2017–2019 (*p* > 0.05). *E. coli* was the most prevalent MDRO-PDAP isolate, with high sensitivity to meropenem (96.0%) and piperacillin/tazobactam (89.1%). *Staphylococcus aureus* was the second most common isolate and was susceptible to vancomycin (100%) and linezolid (100%). Compared to non-multidrug-resistant organism-PDAP, MDRO-PDAP was associated with a lower cure rate (66.4% vs. 85.5%), higher relapse rate (16.4% vs. 8.0%), and higher treatment failure rate (17.1% vs.6.5%). Dialysis age [odds ratio (OR): 1.034, 95% confidence interval (CI): 1.016–1.052, *p* < 0.001] and >2 previous peritonitis episodes (OR: 3.400, 95% CI: 1.014–11.400, *p* = 0.047) were independently associated with treatment failure. Furthermore, longer dialysis age (OR: 1.033, 95% CI: 1.003–1.064, *p* = 0.031) and lower blood albumin level (OR: 0.834, 95% CI: 0.700–0.993, *p* = 0.041) increased the risk of therapeutic failure for MDR-*E. coli* infection.

**Conclusion:**

The proportion of MDRO-PDAP has remained high in recent years. MDRO infection is more likely to result in worse outcomes. Dialysis age and previous multiple peritonitis infections were significantly associated with treatment failure. Treatment should be promptly individualized based on local empirical antibiotic and drug sensitivity analyses.

## 1. Introduction

The incidence of end-stage renal disease (ESRD) has been on the rise globally (1). Peritoneal dialysis (PD) is one of the most common renal replacement therapies; however, peritonitis remains a leading cause of technical failure in PD patients (2). Although prevention and treatment techniques have improved, peritonitis still plays a significant role in the mortality of PD patients (3, 4), and the occurrence of peritonitis has negative impacts on the survival of PD patients (5).

Infections caused by multi-drug resistant (MDR) bacteria are increasing (6), and the emergence of MDR-organism (MDRO) is a serious obstacle in the treatment of PD-associated peritonitis (PDAP). One of the main causes of antimicrobial resistance is the overuse of antibiotics (7). Furthermore, with the continuous adaptation and evolution of bacteria, it is becoming increasingly difficult to rationally choose antibiotics. Therefore, it is necessary to monitor the antimicrobial sensitivity patterns of MDROs to choose antibiotics more wisely.

Presently, studies on MDRO-PDAP are mainly limited to specific bacteria, such as *Acinetobacter* spp. (8), including *Acinetobacter baumannii* (9), and *Corynebacterium striatum* (10), and most of them are case reports or reviews. In previous studies, hypoproteinemia, malnutrition, sex, and diabetes have been identified as risk factors for adverse peritonitis outcomes (11, 12); however, the risk factors for poor MDRO-PDAP outcomes remain unclear. Furthermore, MDRO infection is a global health and economic threat with negative clinical consequences if not recognized and treated adequately (13). In summary, considering the growing problem of MDRO-PDAP, our major objective was to identify patient characteristics, predictors of treatment failure, and microbiological profiles to prevent antibiotic abuse and improve the clinical outcomes of MDRO-PDAP.

## 2. Methods

### 2.1. Patient selection and study design

We retrospectively screened 1,155 patients with peritonitis who underwent PD at the Second Hospital of Jilin University, Jilin Central Hospital, the First Hospital of Jilin University-the Eastern Division, and Jilin FAW General Hospital between January 1, 2013 and December 31, 2019. The inclusion criteria were the PDAP diagnostic criteria issued by the 2022 International Society for Peritoneal Dialysis (ISPD). The exclusion criteria were: (1) patients without complete data, (2) PD fluid that was not cultured, (3) negative PD culture, (4) PD fluid infected by multiple pathogens or fungi, and (5) drug sensitivity results could not be obtained.

Once a patient presented with peritonitis symptoms such as abdominal pain or cloudy dialysis fluid suspected to be caused by PDAP, the specimen of dialysis fluid was collected and sent for microbiological culture and sensitivity test, and then the patients was administered empirical intraperitoneal antibiotics. Most of the empirical treatment regimens were first-generation cephalosporins or vancomycin combined with third-generation cephalosporins or aminoglycoside drugs, and the corresponding antibiotic therapy plan was adjusted after obtaining the bacterial culture and drug sensitivity results. Finally, patients were treated according to the ISPD peritonitis treatment recommendations for 2–3 weeks, and clinicians decided whether to conduct catheter removal.

The study participants were divided into a MDRO group and a non-multidrug-resistant organism (NMDRO) group based on the results of microbial culture and drug sensitivity in the peritoneal dialysate fluid. Combined with patients’ other clinical data, we mainly studied the clinical characteristics, microbiological overview, and risk factors of treatment failure. Additionally, we also analyzed the risk factors for treatment failure of MDR-*Escherichia coli* (*E. coli*)-PDAP specifically. This study was conducted in line with the Declaration of Helsinki. The Ethics Committee of the Second Hospital of Jilin University approved this study; ethics approval number: 2020026. Informed consent was not required due to the retrospective study design.

### 2.2. Data collection

All clinical data were obtained from patients’ record review, including age, dialysis duration, sex, etiology of renal failure, comorbidities, past infections of MDRO-PDAP, number of previous episodes of peritonitis, and laboratory index (for white blood cell count, neutrophil percentage, and neutrophil count in blood, hemoglobin, albumin, potassium, calcium, phosphorus, blood urea nitrogen, serum creatinine, and dialysate white cell count on the first day of PDAP) before or at diagnosis of the index PDAP episode. Treatment outcomes were classified as the initial treatment evaluation, which was primary response and the follow-up treatment evaluation, which included clinical cure, relapse, catheter removal, and PDAP-related death. Drug sensitivity data were also recorded. Finally, we collected the microbiological results of peritoneal dialysate cultures taken from the patients on admission to identify gram-positive, gram-negative, anaerobic bacteria, *Mycobacterium tuberculosis*, fungi, and polymicrobial infections.

### 2.3. Definition

In accordance with the 2022 ISPD guidelines (14), when at least two of the following conditions were present, peritonitis was diagnosed: (1) abdominal pain and/or cloudy ascites, (2) white blood cells in the dialysate >0.1 × 10^9^/L or >100/*μ*L (after at least 2 h dwell time), with >50% polymorphonuclear neutrophil cells, and (3) a positive dialysate pathogen culture. However, the surveillance and study of MDROs has been compromised by the lack of a complete consensus on the definition. Thus, MDRO was considered to be insensitivity to at least one agent in three or more antimicrobial categories following the joint recommendations for epidemiologic studies from the European Center for Disease Prevention and Control (15). The guide also clearly defines common MDROs in healthcare systems, including *Staphylococcus aureus*, *Enterococcus* spp., *Enterobacteriaceae* (other than *Salmonella* and *Shigella*), *Pseudomonas aeruginosa*, and *Acinetobacter* spp. We have only conducted related studies on the above bacteria for reliability and unity of definitions. The effective primary response was identified, when the symptoms of PDAP were significantly alleviated, the dialysate fluid was cleared, and the white blood cell count of the fluid decreased significantly within 48–72 h of reasonable anti-infection treatment (16). Clinical cure implied reasonable antibiotic treatment for 2–3 weeks, complete relief of clinical peritonitis symptoms, clear peritoneal fluid, and white blood cell count in dialysis fluid <0.1 × 10^9^/L (14). PDAP-related death implied death occurring within 30 days of the onset of peritonitis or death due to peritonitis during hospitalization (14). Relapse implied an episode occurring within 4 weeks of therapy completion for a previous episode caused by the same organism (14). Recurrent implied a peritonitis episode occurring within 4 weeks of therapy completion for a previous episode caused by a different organism (14). Repeat implied a peritonitis episode occurring more than 4 weeks after therapy completion for a previous episode caused by the same organism (14). Treatment failure implied catheter removal or PDAP-related death (17).

### 2.4. Statistical analysis

Sample size estimation was performed using PASS version 16.0 (NCCS LLC, Kaysville, Utah, USA), setting a power value of 0.80, an *α* of 0.05, and a ratio of 1:3 for the number of patients in the two groups, yielding a minimum ideal sample size of 139 cases in the MDRO group and 390 cases in the NMDRO group, respectively. All statistical data were analyzed using SPSS version 26.0 (IBM Corp., Armonk, NY, USA). For categorical variables, data were analyzed as frequencies and percentages; Pearson’s chi-square and Fisher’s exact tests were used to compare categorical variables between groups. For continuous variables, data were analyzed as interquartile ranges [M, (P_25_, P_75_)], mean ± standard deviation $\bar{(x}\pm s)$ , using the independent *t*-test (normal distribution) and Wilcoxon rank-sum test (non-normal distribution) to compare continuous variables between groups. Poisson regression was used to compare the incidence of peritonitis. Risk factors influencing treatment failure were analyzed using logistic regression models. Variables with *p*-values <0.05 in the univariate analysis and factors that may influence treatment outcome (age > 60 years, diabetes mellitus) were included in the multivariate model for MDRO-PDAP treatment failure for correction. However, due to limitations in the positive sample size, only variables with *p*-values < 0.05 in the univariate analysis were supported for inclusion in the multivariate model of MDR-*E. coli*-PDAP treatment failure. Statistical significance was set at a *p*-value < 0.05. All probabilities were two-tailed. All figures were plotted using GraphPad Prism version 8.0 (GraphPad Software, San Diego, CA, United States).

## 3. Results

### 3.1. Clinical characteristics

**Figure 1:**
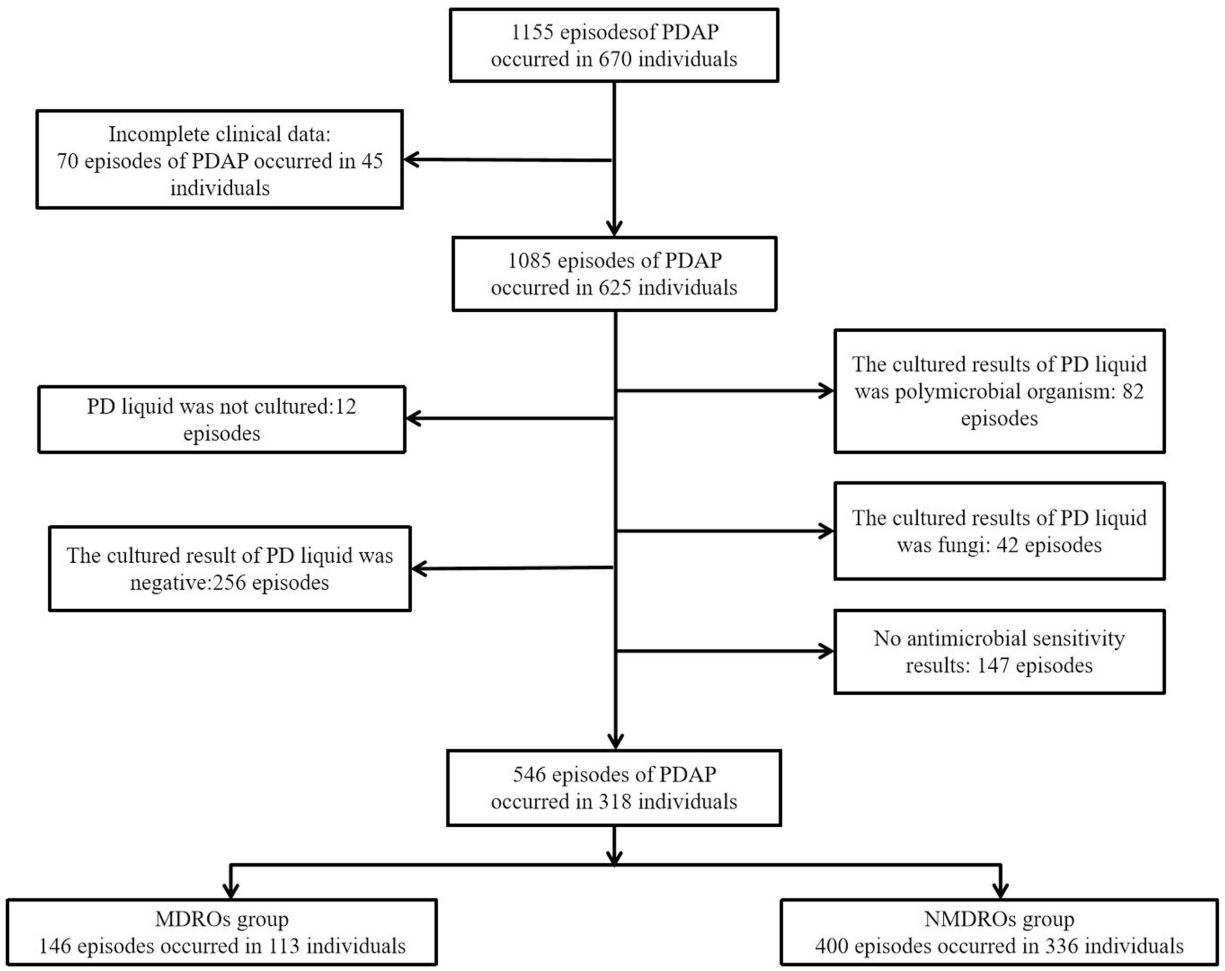
Flow chart. PD, peritoneal dialysis; PDAP, peritoneal dialysis-associated peritonitis; MDRO, multi-drug resistant organism; NMDRO, non-multi-drug resistant organism.

A total of 1,155 PDAP episodes occurred in 670 individuals from January 1, 2013 to December 31, 2019. The screening process for including patients is shown in Figure 1. After applying the exclusion criteria, 318 individuals and 546 cases were finally included in the study, with 113 patients and 146 cases included in the MDRO group while 336 patients and 400 cases were included in the NMDRO group. During the study period, 93 patients experienced only one episode of MDRO-PDAP, 11 patients experienced only two episodes of MDRO-PDAP, 7 patients experienced only three episodes, 1 patient experienced only four episodes, and 1 patient experienced six episodes.

Comparisons of clinical characteristics between the two groups are presented in Table 1. Patients in the MDRO group had a higher percentage of blood neutrophils, were more likely to have past infections of MDRO-PDAP, more likely to show polycystic kidney as protopathy, and were more likely to be relapsing cases (*p* < 0.05) than those in the NMDRO group. Conversely, patients in the NMDRO group had higher blood albumin levels and were more likely to be initial cases than those in the MDRO group (*p* < 0.05). However, additional parameters showed no significant differences between the two groups. The details of each pathogenic bacteria studied are presented in Table 1.

**Table 1:** Comparison of clinical characteristics between the MDRO and NMDRO groups.

Clinical variables	MDRO group (*n* = 146)	NMDRO group (*n* = 400)	*t*/*X*^2^/*Z*	*p*-value	MDRO^a^				
					Staphylococcus aureus (*n* = 34)	Enterobacteriaceae (*n* = 101)	Acinetobacter spp. (*n* = 7)	Pseudomonas aeruginosa^b^ (*n* = 2)	Enterococcus spp.^b^ (*n* = 2)
Age [year, (M, P_25_, P_75_)]	62.0 (48.0, 70.0)	58.0 (46.0, 68.0)	2.632	0.105	61.0 (47.8, 66.0)	63.0 (48.0, 70.0)	67.5 (47.8, 79.5)	58.5	71.5
Gender [men (*n*, %)]	78 (53.4%)	210 (52.5%)	0.037	0.848	22 (64.7%)	49 (48.5%)	6 (85.7%)	1 (50.0%)	0 (0%)
Dialysis age [month, (M, P_25_, P_75_)]	17.5 (4.0, 33.5)	16.0 (7.0, 30.0)	0.133	0.715	13.5 (4.8, 22.3)	20.0 (5.0, 46.0)	20.0 (1.5, 44.8)	3.5	1.5
Past infection of MDRO (*n*, %)	33 (22.6%)	21 (14.4%)	36.140	<0.001	12 (35.3%)	19 (18.8%)	0 (0%)	0 (0%)	0 (0%)
Protopathy									
Chronic glomerulonephritis (*n*, %)	62 (42.5%)	153 (38.3%)	0.796	0.372	12 (35.3%)	48 (47.5%)	1 (14.3%)	1 (50.0%)	0 (0%)
Diabetic nephropathy (*n*, %)	15 (10.3%)	66 (16.5%)	3.282	0.070	16 (47.1%)	17 (16.8%)	1 (14.3%)	0 (0%)	1 (50.0%)
Interstitial nephritis (*n*, %)	9 (6.1%)	12 (3%)	2.896	0.089	2 (5.9%)	7 (6.9%)	0 (0%)	1 (50.0%)	0 (0%)
Hypertensive nephropathy (*n*, %)	35 (24.0%)	110 (27.5%)	0.682	0.409	2 (5.9%)	11 (10.9%)	1 (14.3%)	0 (0%)	1 (50.0%)
Polycystic kidney (*n*, %)	13 (8.8%)	17 (4.3%)	4.462	0.035	1 (2.9%)	17 (16.8%)	1 (14.3%)	0 (0%)	0 (0%)
Accompanying disease									
Hypertension (*n*, %)	119 (81.5%)	351 (87.8%)	3.479	0.062	30 (88.2%)	82 (81.2%)	4 (57.1%)	1 (50.0%)	2 (100.0%)
Diabetes (*n*, %)	45 (30.8%)	157 (39.2%)	3.260	0.071	16 (47.1%)	27 (26.7%)	1 (14.3%)	0 (0%)	0 (0%)
Laboratory index									
WBC count [*10^9/L, (M, P_25_, P_75_)]	8.26 (6.25, 12.16)	8.46 (6.29, 11.39)	0.031	0.860	8.25 (6.62, 12.37)	8.23 (5.93, 11.97)	8.75 (5.63, 18.44)	15.63	12.41
NEU percentage [%, (M, P_25_, P_75_)]	85.95 (79.55–90.65)	82.40 (75.01–88.0)	12.327	<0.001	87.50 (79.91, 91.23)	85.40 (79.50, 90.15)	83.25 (81.30, 91.55)	85.57	74.15
NEU count [*10^9/L, (M, P_25_, P_75_)]	7.34 (4.87–10.20)	6.92 (4.72–9.85)	0.935	0.334	7.38 (5.60, 10.52)	7.20 (4.71, 10.12)	7.47 (4.62, 17.05)	13.67	9.66
Hb [g/L, ( x_±s )]	100.33 ± 17.05	98.16 ± 20.13	−1.249	0.213	89.97 ± 12.14	104.29 ± 17.13	89.00 (74.50, 109.00)	94.50	104.00
Alb [g/L, ( x_±s )]	27.83 ± 6.38	29.21 ± 5.90	2.362	0.019	26.86 ± 7.25	27.98 ± 5.78	31.49 ± 10.13	14.55	22.55
Potassium [mmol/L, (M, P_25_, P_75_)]	3.74 (3.30, 4.24)	3.79 (3.30, 4.27)	0.086	0.770	3.91 (3.53, 4.68)	3.71 (3.24, 4.20)	3.5 (3.04, 3.83)	3.33	3.17
Calcium [mmol/L, (M, P_25_, P_75_)]	2.11 (1.98, 2.29)	2.17 (2.01, 2.31)	2.422	0.120	2.03 (1.90, 2.28)	2.13 (2.00, 2.30)	2.11 (2.06, 2.42)	2.35	1.94
Phosphorus [mmol/L, (M, P_25_, P_75_)]	1.22 (0.99, 1.51)	1.28 (1.05, 1.55)	2.448	0.118	1.26 (1.07, 1.54)	1.22 (0.97, 2.30)	1.05 (0.79, 1.70)	1.28	1.41
BUN [mmol/L, (M, P_25_, P_75_)]	25.44 (11.61, 20.73)	15.88 (12.02, 19.84)	0.081	0.777	16.21 (12.34, 24.87)	14.77 (11.39, 20.44)	17.06 (14.96, 18.21)	12.84	23.17
Scr [μmol/L, (M, P_25_, P_75_)]	707.94 (529.68, 907.31)	738 (543.13, 920.03)	0.884	0.347	729.21 (578.85, 829.42)	703.30 (528.45, 910.50)	708.37 (335.71, 1047.83)	522.77	430.91
Nature of peritonitis									
Initial PDAP (*n*, %)	75 (51.4%)	244 (61.0%)	4.084	0.043	18 (52.9%)	47 (46.5%)	6 (85.7%)	2 (100.0%)	2 (100.0%)
Relapsing PDAP (*n*, %)	17 (11.6%)	26 (6.5%)	3.901	0.048	7 (20.6%)	10 (9.9%)	0 (0%)	0 (0%)	0 (0%)
Recurrent PDAP (*n*, %)	9 (6.2%)	20 (5.0%)	0.288	0.591	1 (2.9%)	8 (7.9%)	0 (0%)	0 (0%)	0 (0%)
Repeat PDAP (*n*, %)	9 (6.2%)	30 (7.5%)	0.288	0.592	4 (11.8%)	5 (5.0%)	0 (0%)	0 (0%)	0 (0%)

WBC, white blood cell; NEU, neutrophil; Hb, hemoglobin; Alb, albumin; BUN, blood urea nitrogen; Scr, serum creatinine; PDAP, peritoneal dialysis-associated peritonitis; MDRO, multi-drug resistant organism; NMDRO, non-multi-drug resistant organism.

a In addition to describing the differences between the two groups, we also presented baseline information for each type of MDRO we studied for reference.

b Because there were only two cases of this bacterium, the continuous variables were described using the mean.

### 3.2. Constituent ratio of MDRO-PDAP and pathogen distribution

**Figure 2:**
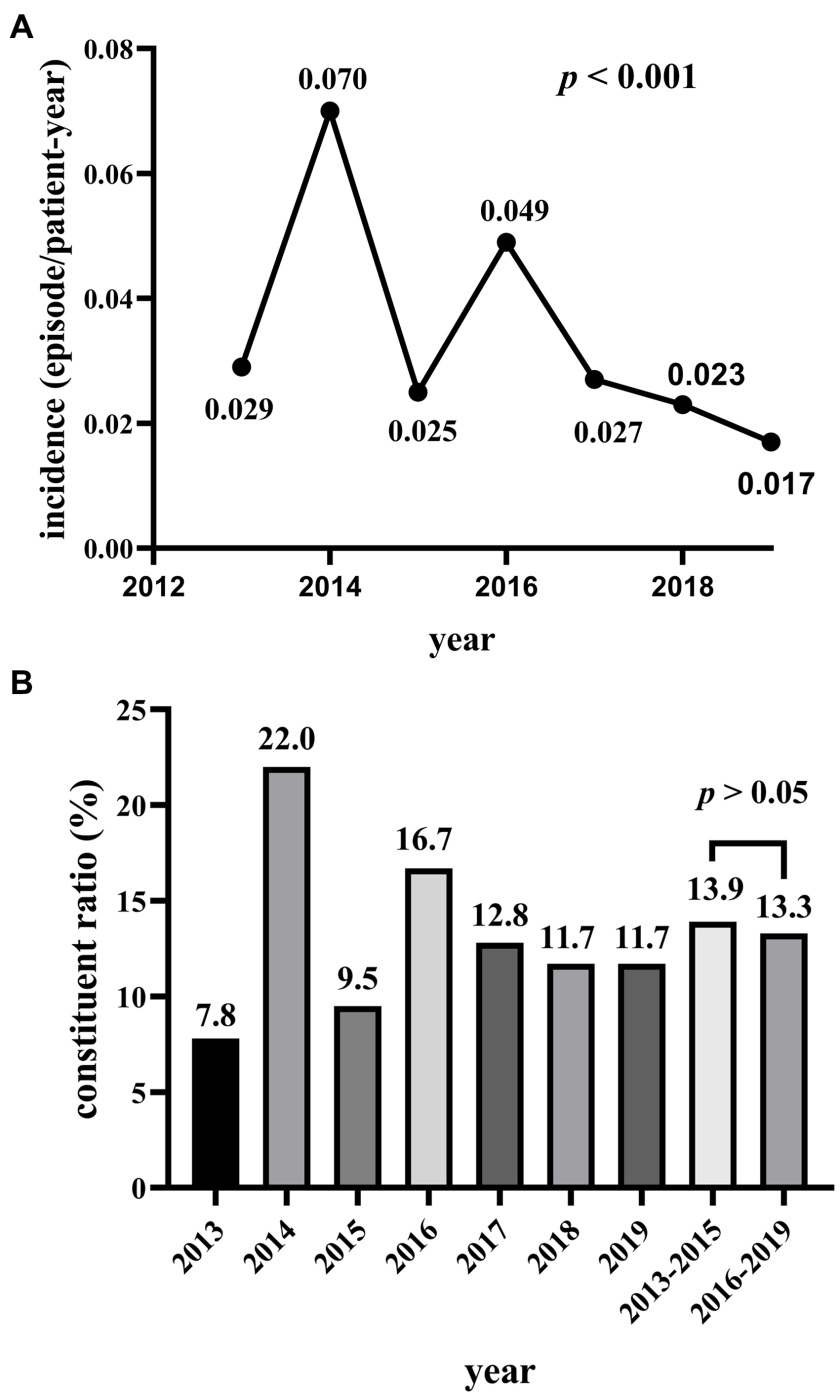
**(A)** The incidence of MDRO-PDAP from 2013 to 2019. **(B)** The constituent ratio of MDRO-PDAP from 2013 to 2019.

A total of 146 cases of MDRO-PDAP accounted for 12.6% of the total 1,155 cases of PDAP over seven years. From 2013 to 2019, the average incidence of peritonitis was 0.225 episodes/patient-year, and the overall trend in incidence of MDRO-PDAP was decreasing (*p* < 0.001; Figure 2A); however, the composition ratio of MDRO-PDAP remained high from 2013–2015 (13.9%) to 2016–2019 (13.3%) (*p* > 0.05; Figure 2B). A total of 146 MDRO strains were isolated from 546 PDAP cases. Among these MDROs, gram-negative bacterial isolates (*n* = 110, 75.3%) were more common than gram-positive bacterial isolates (*n* = 36, 24.7%). MDR-*E. coli* (*n* = 79, 54.1%), followed by *Klebsiella pneumoniae* (*n* = 10, 6.8%), was the most common antimicrobial isolate among gram-negative strains, accounting for 78.2% (79/92) of all *E. coli* isolates. MDR-*S. aureus* (*n* = 34, 23.3%), followed by *Enterococcus* (*n* = 2, 1.4%), was the most common isolate among the gram-positive strains, accounting for 73.9% (34/46) of all *S. aureus* isolates (Table 2).

**Table 2:** The distribution of causative organisms in patients infected with MDRO.

Pathogenic microorganisms	*n* (%)
Gram-positive bacteria	36 (24.7%)
*Staphylococcus aureus*	34 (23.3%)
*Enterococcus* spp.	2 (1.4%)
Gram-negative bacteria	110 (75.3%)
*Enterobacteriaceae*	101 (69.2%)
*Escherichia coli*	79 (54.1%)
*Klebsiella pneumoniae*	10 (6.8%)
*Others*	12 (8.2%)
*Pseudomonas aeruginosa*	2 (1.3%)
*Acinetobacter* spp.	7 (4.8%)
*Baumannii*	4 (2.7%)
*Others*	3 (2.1%)
Total	146 (100%)

MDRO, multi-drug resistant organism.

### 3.3. Antimicrobial susceptibility analysis

**Figure 3:**
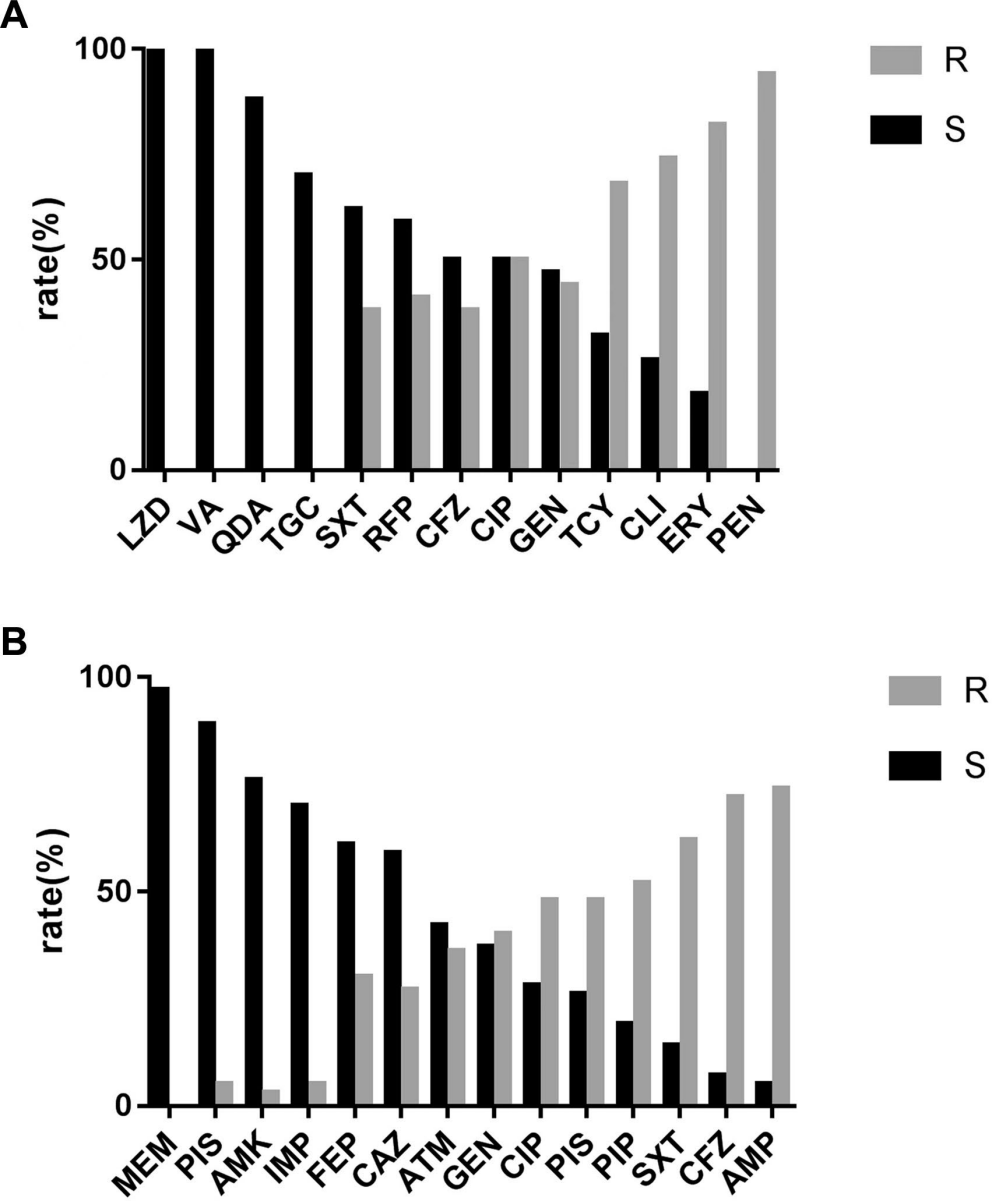
**(A)** Antimicrobial sensitivity result of MDR-*Staphylococcus aureus*. LZD, linezolid; VA, vacomycin; QDA, quinupristin-dalfopristin; TGC, tigecycline; SXT, trimethoprim-sulphamethoxazole; RFP, rifampicin; CFZ, cephazolin; CIP, ciprofloxacin; GEN, gentamicin; TCY, tetracucline; CLI, clindamyxin; ERY, erythrocin; PEN, penicillin. **(B)** Antimicrobial sensitivity result of MDR-*Escherichia coli* MEM, meropenem; PIS, piperacillin/tazobactam; AMK, amikacin; IMP, imipenem; FEP, cefepime; CAZ, ceftazidime; ATM, aztreonam; GEN, gentamicin; CIP, ciprofloxacin; SAM, ampicillin/sulbactam; PIP, peperacillin; SXT, trimethoprim-sulphamethoxazole; CFZ, cephazolin; AMP, ampicillin.

Figures 3A,B show the antibiotic sensitivity results with the two MDROs (*S. aureus* and *E. coli*) representing the largest proportion. All *S. aureus* strains showed susceptibility to vancomycin and linezolid (Figure 3A). The *enterococcus* spp. isolates were 100% susceptible to linezolid. Among gram-negative bacteria, *E. coli* strains were highly susceptible to meropenem (96.0%) and piperacillin/tazobactam (89.1%; Figure 3B). *P. aeruginosa* and *Acinetobacter* spp. were completely susceptible to meropenem and imipenem, respectively.

### 3.4. Evaluation of treatment outcome

Table 3 shows the treatment outcomes of the two groups. Compared to the NMDRO group, the MDRO group had a lower initial effective rate (70.5% vs. 88.5%, *p* < 0.001), lower cure rate (66.4% vs. 85.5%), higher relapse rate (16.4% vs. 8.0%), and higher treatment failure rate (17.7% vs. 6.5%, *p* < 0.001), which included a higher frequency of catheter removal (11.6% vs. 3.8%) and death (5.5% vs. 2.8%).

**Table 3:** Evaluation of treatment between MDRO group and NMDRO group.

Therapeutic evaluation	MDRO group (*n* = 146)	NMDRO group (*n* = 400)	*X* ^2^	*p*-value	MDRO^a^				
					Staphylococcus aureus (*n* = 34)	Enterococcus spp. (*n* = 101)	Acinetobacter spp. (*n* = 7)	Pseudomonas aeruginosa (*n* = 2)	Enterobacteriaceae (*n* = 2)
Effective initial treatment (*n*, %)	103 (70.5%)	354 (88.5%)	25.265	<0.001	28 (82.4%)	67 (66.3%)	5 (71.4%)	2 (100.0%)	1 (50.0%)
Evaluation of follow-up treatment			25.921	<0.001	–	–	–	–	–
Cure (*n*, %)	97 (66.4%)	342 (85.5%)	–	–	22 (64.7%)	68 (67.3%)	5 (71.4%)	1 (50.0%)	1 (50.0%)
Relapse (*n*, %)	24 (16.4%)	32 (8.0%)	–	–	8 (23.5%)	14 (13.9%)	1 (14.3%)	0 (0%)	1 (50.0%)
Catheter removal (n, %)	17 (11.6%)	15 (3.8%)	–	–	4 (11.8%)	12 (11.9%)	1 (14.3%)	0 (0%)	0 (0%)
Death associated with peritonitis (*n*, %)	8 (5.5%)	11 (2.8%)	–	–	0 (0%)	7 (6.9%)	0 (0%)	1 (50.0%)	0 (0%)
Treatment failure ^b^	25 (17.1%)	26 (6.5%)	14.254	<0.001	4 (11.8%)	19 (18.8%)	1 (14.3%)	1 (50.0%)	0 (0%)

MDRO, multi-drug resistant organism; NMDRO, non-multi-drug resistant organism.

a In addition to describing the differences between the two groups, we also presented baseline information for each type of MDRO we studied for reference.

b Treatment failure including catheter removal and peritonitis-related death.

### 3.5. Risk factors for treatment failure

PD age and more than 2 previous PDAP episodes were suggested to be significant in the univariate analysis, and after adjusting for other confounding factors (age > 60 years, diabetes), a multifactorial logistic regression model identified the two independent risk factors for MDRO-PDAP treatment failure, namely PD age [odds ratio (OR): 1.034, 95% confidence interval (CI): 1.016–1.052, *p* < 0.001] and more than 2 previous PDAP episodes (OR: 3.400, 95% CI: 1.014–11.400, *p* = 0.047; Table 4). In addition, we also analyzed the risk factors for MDR-*E. coli*-PDAP treatment failure, with albumin levels and PD age showing significance in the univariate analysis. Putting the above indicators into a multifactorial regression analysis, it was determined that lower albumin levels (OR: 0.834, 95% CI: 0.700–0.993, *p* = 0.041) and PD age (OR: 1.033, 95% CI: 1.003–1.064, *p* = 0.031) increase the risk of MDR-*E. coli*-PDAP treatment failure (see Table 5).

**Table 4:** Risk factors of treatment failure in patients with MDROs group.

Variables	Univariate			Multivariate		
	OR	95%CI	*p*-value	OR	95%CI	*p*-value
Age>60 (years)	1.061	0.446–2.524	0.894	1.105	0.429–2.846	0.836
Male	0.882	0.371–2.099	0.777	–	–	–
Dialysis age (years)	1.034	1.016–1.052	0.000	1.034	1.016–1.052	<0.001
>2 previous peritonitis episodes	3.158	1.043–9.558	0.042	3.400	1.014–11.400	0.047
**Accompanying disease**						
Hypertension	1.232	0.386–3.937	0.725	–	–	–
Diabetes	0.849	0.327–2.205	0.737	0.780	0.269–2.261	0.836
**Laboratory index**						
NEU percentage (%)	1.026	0.974–1.080	0.339	–	–	–
NEU count (*109/L)	0.999	0.920–1.085	0.977	–	–	–
WBC count (*109/L)	0.992	0.915–1.075	0.768	–	–	–
Hb (g/L)	1.004	0.979–1.029	0.768	–	–	–
Alb (g/L)	0.956	0.891–1.026	0.214	–	–	–
Potassium (mmol/L)	0.915	0.511–1.637	0.764	–	–	–
Phosphorus (mmol/L)	0.655	0.259–1.653	0.370	–	–	–
Calcium (mmol/L)	2.659	0.457–15.457	0.276	–	–	–
**Nature of peritonitis**	–	–	0.685	–	–	–
Relapsing	0.712	0.251–2.203	0.524	–	–	–
Repeating	1.275	0.317–5.127	0.732	–	–	–
Recurrent	2.071	0413–10.395	0.376	–	–	–
Initial	0.000	0.000−	0.999	–	–	–
Others	Reference					
**Bacteria**	–	–	0.712	–	–	–
*Staphylococcus aureus*	Reference					
*Enterococcus* spp.	1.738	0.547–5.524	0.349	–	–	–
*Enterobacteriaceae*	0.000	0.000−	0.999	–	–	–
*Pseudomonas aeruginosa*	7.500	0.388–144.973	0.182	–	–	–
*Acinetobacter* spp.	1.250	0.118–13.240	0.853	–	–	–
**Protopathy**	–	–	0.804	–	–	–
Chronic glomerulonephritis	2.115	0.245–188.271	0.496	–	–	–
Diabetic nephropathy	2.750	0.248–30.512	0.410	–	–	–
Interstitial nephritis	5.500	0.464–65.162	0.177	–	–	–
Hypertensive nephropathy	2.276	0.245–21.120	0.469	–	–	–
Polycystic kidney disease	2.000	0.157–25.404	0.593	–	–	–
Others	Reference					
Dialysate white cell count,1st day of PDAP (*10^6^)	1.000	1.000–1.000	0.716	–	–	–

WBC, white blood cell; NEU, neutrophil; Hb, hemoglobin; Alb, albumin; BUN, blood urea nitrogen; Scr, serum creatinine; PDAP, peritoneal dialysis-associated peritonitis; MDRO, multi-drug resistant organisms; OR, odds ratio; 95% CI, 95% confidence interval.

**Table 5:** Risk factors of treatment failure in patients with MDR-*E. coli-*PDAP.

Variables	Univariate			Multivariate		
	OR	95%CI	*p*-value	OR	95%CI	*p*-value
Age > 60 (years)	0.750	0.173–3.249	0.701	–	–	–
Male	1.784	0.413–7.706	0.438	–	–	–
Dialysis age (years)	1.036	1.006–1.067	0.018	1.033	1.003–1.064	0.031
Previous peritonitis episodes	0.552	0.937–2.570	0.088	–	–	–
**Accompanying disease**						
Hypertension	0.798	0.148–4.296	0.793	–	–	–
Diabetes	0.767	0.14604.023	0.754	–	–	–
**Laboratory index**						
NEU percentage (%)	1.046	0.942–1.077	0.339	–	–	–
NEU count (*109/L)	1.007	0.942–1.071	0.832	–	–	–
WBC count (*109/L)	1.047	0.939–1.169	0.408	–	–	–
Hb (g/L)	0.997	0.957–1.039	0.892	–	–	–
Alb (g/L)	0.841	0.719–0.983	0.030	0.834	0.700–0.993	0.041
Potassium (mmol/L)	0.796	0.315–2.012	0.623	–	–	–
Phosphorus (mmol/L)	0.716	0.189–2.709	0.623	–	–	–
Calcium (mmol/L)	1.275	0.070–23.121	0.869	–	–	–
**Nature of peritonitis**	–	–	0.881	–	–	–
Relapsing	0.463	0.094–2.278	0.344	–	–	–
Repeating	0.875	0.082–9.376	0.912	–	–	–
Recurrent	1.313	0.115–15.032	0.827	–	–	–
Initial	0.000	0.000−	0.999	–	–	–
Others	Reference					
**Protopathy**	–	–	0.727	–	–	–
Chronic glomerulonephritis	Reference					
Diabetic nephropathy	2.188	0.339–14.095	0.410	–	–	–
Interstitial nephritis	4.375	0.599–31.934	0.146	–	–	–
Hypertensive nephropathy	0.729	0.074–7.181	0.787	–	–	–
Polycystic kidney disease	0.000	0.000−	0.999	–	–	–
Others	0.000	0.000−	0.999	–	–	–
Dialysate white cell count,1st day of PDAP (*10^6^)	1.000	1.000–1.000	0.557	–	–	–

WBC, white blood cell; NEU, neutrophil; Hb, hemoglobin; Alb, albumin; BUN, blood urea nitrogen; Scr, serum creatinine; PDAP, peritoneal dialysis-associated peritonitis; MDRO, multi-drug resistant organisms; OR, odds ratio; 95% CI, 95% confidence interval.

## 4. Discussion

To our knowledge, this is the first multicenter retrospective study to explore the clinical characteristics, antimicrobial susceptibility, patient outcomes, and risk factors of treatment failure associated with MDRO-PDAP.

The average peritonitis rate was 0.225 episodes/patient-year in our study from 2013 to 2019, and per the ISPD recommendations updated in 2022, the requirement is no more than 0.4 episodes per patient-year, indicating that the incidence of peritonitis was relatively well controlled in the hospitals where this study was conducted. Another study from our center pointed out that the incidence of PDAP has decreased in recent years (18), and our study also observed that the incidence of MDRO-PDAP was similarly decreasing between 2013–2019. However, MDRO-PDAP remains a serious challenge for clinicians because of increasing drug resistance among bacteria. Considering that MDRO-PDAP incidence could be influenced by the overall incidence of peritonitis, we further studied the composition ratio of MDRO-PDAP in all PDAP cases. Not surprisingly, we observed that the proportion of MDRO-PDAP remained high in these years. During the study period, 93 patients with MDROs experienced only one episode of MDRO-PDAP, 11 patients experienced only two episodes, and 9 patients experienced more than two episodes. These results support the notion that, because of the seriousness of the problem presented by MDRO, the vigilance for MDROs should be improved and further research conducted on the microbiological profiles and risk factors of MDRO-PDAP to fill the current gap in research.

We observed that the percentage of neutrophils was higher in the MDRO group than in the NMDRO group, with higher expression levels of inflammatory mediators, resulting in a stronger inflammatory response and a more severe degree of disease in the MDRO group; this finding is supported by an article demonstrating elevated expression of inflammatory mediators in endophthalmitis patients infected with MDR-*P. aeruginosa* (19). Another study (20) from China clarified that elevated neutrophil levels are a sensitive systemic inflammatory marker associated with high mortality in patients with ESRD. Furthermore, several studies (21, 22) have associated hypoalbuminemia with the development of PDAP. In our study, the MDRO group had low albumin levels. Serum albumin level is often used to assess patient nutritional condition, although hypoalbuminemia may also be linked to inflammation. Malnutrition affects immunity and causes immune dysfunction, which in turn affects the resistance to infection. Besides, some studies (13, 23) have noted that having a history of MDROs is associated with positive MDRO isolation on admission; this could partially explain why the MDRO group in our study were more likely to have a history of MDRO colonization.

Compared to the NMDRO group, the MDRO group had more relapsing cases, which required medical professionals to pay closer attention to patients with relapsing peritonitis because they are more prone to infections by MDROs. A systematic review (24) indicated that previous antibiotic use was related to the likelihood of MDRO isolation, possibly because prior antimicrobial treatment strongly modified the abdominal microbiota and was associated with an increased risk of drug-resistant microbial infection. Interestingly, there were no significant differences in the recurrent and repeat cases between the case and control groups. Besides, the MDRO group had a lower initial treatment effective rate and higher treatment failure rate compared with the NMDRO group. Reportedly, a poor prognosis was more common in patients with peritonitis due to methicillin-resistant *S. aureus* (MRSA), vancomycin-resistant *enterococci*, and extended spectrum β-lactamase (ESBL)- and metallo-β-lactamase-producing bacteria (25, 26), which supported our conclusion to some extent that MDRO-PDAP tended to have poor outcomes. Therefore, it is necessary to adjust the poor treatment plan of MDRO-PDAP on the basis of the drug sensitivity results and attempt to reduce the related risk to prevent poor prognosis whenever possible.

In our multivariate study, PD age and more than 2 previous PDAP episodes were independently associated with MDRO-PDAP treatment failure. Our previous study showed that long dialysis age was a risk factor for treatment failure of first peritonitis (27). The thickness of the submesothelial layer increases gradually and is accompanied by peritoneal fibrosis and neovascularization in the peritoneum of patients with long-term PD. Thus, these patients have a higher incidence of ultrafiltration failure (28). In this study, we observed a 2.4-fold increase in the risk-of-failure of the current peritonitis treatment when there were more than 2 previous PDAP episodes. Repeated episodes of peritonitis cause aggregation of inflammatory cells such as mononuclear macrophages and release transforming growth factor-β (TGF-β), a major molecule in the course of peritoneal fibrosis, and the overexpression of TGF-β is linked to worse PD outcomes (29). We further inferred that the effect of empirical antimicrobial therapy could be compromised based on the above pathological basis if the patient had an infection caused by MDROs, with treatment delay increasing the likelihood of treatment failure. In our study, non-first-episode peritonitis with the same pathogen cultured in the previous peritonitis episode accounted for 74.3% (26/35) of all non-first-episode peritonitis cases. This also suggests that in patients who are repeatedly hospitalized for peritonitis in a short period, clinicians should choose the initial treatment regimen in conjunction with their previous culture and drug susceptibility results. There are few studies related to the risk factors for treatment failure in MDR-*S. aureus* and MDR-*E. coli*; however, to avoid statistical bias caused by the few cases of *S. aureus* treatment failure, we only studied *E. coli*, which is the most prevalent species in the *Enterobacteriaceae* family. We observed that lower albumin levels and longer PD age increased the risk of treatment failure, similar to the results of another study (30) conducted in our center on the treatment outcome of *E. coli* infection. Therefore, for *E. coli*-PDAP, especially those infected with MDROs, it could be beneficial for clinical outcomes to raise albumin levels.

Previous studies (31, 32) showed that the most common microorganisms isolated from patients with PDAP were gram-positive bacteria, among which coagulase-negative *Staphylococcus* was the most prevalent, followed by *S. aureus*. Furthermore, *E. coli* was the most common gram-negative bacterium, followed by *Klebsiella pneumoniae* and *P. aeruginosa*. However, because coagulase-negative *Staphylococcus* was not included in the MDRO group in our study, the microbiological distribution of MDROs in our study was slightly different from the results of the PDAP population. Therefore, as shown in Table 2, gram-positive MDROs were mainly *S. aureus* and *E. coli*, followed by *Klebsiella pneumoniae*, and *Baumannii* was the most common gram-negative bacterium.

Owing to the small number of strains of other MDROs, including *P. aeruginosa*, *Enterococcus* spp., and *Acinetobacter* spp., we did not perform detailed drug susceptibility analysis of the above pathogens to avoid a small sample size distorting study results but instead focused on MDR-*S. aureus* and MDR-*E. coli*. The causative organism that produced more severe outcomes in MDR-*S. aureus* PDAP was MRSA, which are usually more severe than peritonitis caused by methicillin-susceptible *S. aureus*, with significantly increased frequency of hospitalization and length of treatment time, as well as increased frequency of extubation and death (33). Severe negative outcomes were more likely to occur when vancomycin was not used in the regimen of treatment for MRSA peritonitis (33). In our study, all the MDR-*S. aureus* strains were susceptible to vancomycin, reflecting the importance of vancomycin in the treatment of MDR-*S. aureus*-infection. Producing extended-spectrum beta-lactamases (ESBLs) is the most vital resistance mechanism in *Enterobacteriaceae*. In a study of MDROs from Hong Kong (6), *E. coli* accounted for 85.6% of all ESBL-producing isolates. Unfortunately, owing to the limitations of this retrospective study, ESBL-producing organisms could not be accurately detected. Carbapenems are ideal therapeutic agents against this bacterium (34). The 2022 ISPD guidelines also state that for *Enterobacteriaceae*, treatment regimens should be based on resistance patterns. Intraperitoneal application of meropenem could be an option for ESBL-producing Enterobacteriaceae (ESBL-E) (14). The treatment recommendations were also supported by our finding that almost all MDR-*E. coli* cultured *in vitro* are susceptible to meropenem. However, it is worth noting that except for ESBL-E, *Enterobacteriaceae* had evolved to resist carbapenems (6), causing mortality rates of up to 70% (35) which are rising globally (36). Above all, our results showed that the local antimicrobial susceptibility patterns of MDR-*E. coli* and MDR-*S. aureus* were consistent with the therapeutic antibiotics recommended by the guidelines. Nevertheless, the final treatment plan should still be carefully selected according to the drug susceptibility results to prevent more resistance caused by antibiotic abuse.

This study had some limitations. Firstly, the retrospective nature of the study resulted in the presence of some unavoidable biases. For example, the results of this study cannot be extrapolated to the general population because of the confined location from which patients were included and the treatment regimens in each region. What’s more, we excluded cases with mixed bacterial infections due to concerns that they would interfere with the analysis of treatment failure of MDR-PDAP, but mixed infections are more likely to develop antibiotic resistance than isolated infections (37), which might introduce selection bias. Secondly, we were unable to identify MRSA or ESBL-E based on the collected drug sensitivity results; therefore, our efforts to analyze the antibiotic sensitivity pattern in more detail were limited. Finally, the number of MDROs for some species was too few to be analyzed categorically, so larger prospective studies are warranted in the future.

## 5. Conclusion

In summary, MDRO-PDAP has remained a major issue in recent years, resulting in poor treatment outcomes. Special attention should be paid to patients with more than two previous peritonitis episodes, and those who have been undergoing dialysis for a long time because treatment for such patients is more likely to fail. Although the treatment regimens recommended by the current guidelines for infections caused by MDR-*S. aureus* and MDR-*E. coli* are appropriate in the area where we are conducting our study; they should be modified promptly in accordance with drug susceptibility to reduce the adverse outcomes caused by delays in MDRO-PDAP treatment.

## Data availability statement

The raw data supporting the conclusions of this article will be made available by the authors, without undue reservation.

## Ethics statement

The studies involving human participants were reviewed and approved by the Ethics Committee of Second Hospital of Jilin University (No. 2020026). The ethics committee waived the requirement of written informed consent for participation.

## Author contributions

LMY, XYZ, and XXZ provided the data. LFM, XYL, SYC, and XHZ collected the data. SZG performed the statistical analysis and wrote the manuscript. WPC and SML designed the study and reviewed this manuscript. All authors contributed to the article and approved the submitted version.

## Funding

This study was supported by the Research Project of the Branch of Blood Purification Center of the Chinese Hospital Association (Project No. CHABP 2021-20) and the Science and Technology department of Jilin Province (Project No. YDZJ202201ZYTS110).

## Conflict of interest

The authors declare that the research was conducted in the absence of any commercial or financial relationships that could be construed as a potential conflict of interest.

## Publisher’s note

All claims expressed in this article are solely those of the authors and do not necessarily represent those of their affiliated organizations, or those of the publisher, the editors and the reviewers. Any product that may be evaluated in this article, or claim that may be made by its manufacturer, is not guaranteed or endorsed by the publisher.
